# Floral Traits, Pollination and Reproductive Differentiation in Gynodioecious *Minuartia nifensis* (Caryophyllaceae)

**DOI:** 10.3390/plants15060913

**Published:** 2026-03-16

**Authors:** Volkan Eroğlu, Serdar Gökhan Şenol

**Affiliations:** Department of Biology, Science Faculty, Ege University, Bornova, İzmir 35100, Turkey; serdar.gokhan.senol@ege.edu.tr

**Keywords:** gynodioecy, reproduction, conservation, *Minuartia*

## Abstract

The endemic *Minuartia nifensis*, the only known gynodioecious species of its genus, offers a suitable model for understanding the relationships between floral characteristics, pollination, and mating systems in species with narrow distributions and single populations. We analyzed population structure, floral morphology, pollen viability, stigma receptivity, mating system components, and pollinator assemblages using field observations, morphometric measurements, controlled pollination experiments (autogamy, allogamy, apomixis and open pollination), and standardized pollinator surveys. The population exhibited an approximately balanced hermaphrodite–female ratio (0.97:1) and clear sexual dimorphism, with hermaphrodite flowers significantly larger than female flowers. Despite this dimorphism, pollinator visitation was similar between morphs, with 52.54% of visits to hermaphrodite flowers and 47.46% to female flowers. A total of 1734 visits by seven visitor species were recorded, of which approximately 95% of potentially effective pollen transfer was attributable to three bee taxa. Pollen viability, stigma receptivity, and visitation frequency peaked between 12:00 and 14:00, accounting for 58% of total insect visits. Controlled pollination experiments showed highest reproductive success under cross-pollination and limited success under self-pollination, indicating a mixed but predominantly outcrossing mating system. Together, these results suggest that gynodioecy in *M. nifensis* may be supported by floral differentiation, temporal reproductive traits, and pollinator-mediated pollen transfer.

## 1. Introduction

Sexual systems and variations in sex expression are central to angiosperm reproductive systems and have significant evolutionary implications. Polymorphisms such as dioecy, monoecy, and heterostyly promote genetic diversity, influence mating patterns, and drive the evolution of sex chromosomes and sexual dimorphism [1,2,3,4,5,6,7]. Ultimately, these polymorphisms reduce self-fertilization and increase genetic diversity [2,8]. Gynodioecy, defined as the presence of both female and hermaphrodite individuals in the same population [9,10,11,12,13], is a rarity among angiosperms, with a documented prevalence of less than 1%, most of which are members of the Asteraceae family [10,12,13,14]. However, it is estimated to occur phylogenetically in 7–10% of angiosperm families [15,16], or ~21% [17]. Gynodioecy is generally considered an intermediate or stable state in the evolution from hermaphroditism to dioeciousness [11,18]. Mathematical models show that low pollen transport efficiency supports dioecy, while increased efficiency allows for the emergence of hermaphrodites, leading first to trioecy and then to gynodioecy, which reverts back to hermaphroditism as the number of females decreases [19].

Gynodioecy results in distinct and predictable differences in flower morphology between female and hermaphrodite plants. These differences often involve reduced size and increased asymmetry in female flowers, along with sterile or vestigial stamens, patterns that reflect both developmental constraints and evolutionary pressures related to reproduction and pollination [20,21,22,23,24,25]. Hermaphrodite individuals face stronger selection pressures to produce larger, more attractive flowers to guarantee visits from pollinators, while females may invest more in ovule and fruit production [13,22,24,26,27,28,29,30].

Many gynodioecious species use a generalist pollination strategy, which attracts a variety of pollinators and ensures reproductive success under different conditions [31,32,33]. Effective pollen transfer depends on pollinators moving between male, hermaphrodite, and female flowers [12,34,35]. The characteristics of flowers and the behaviors of pollinators together determine reproductive outcomes [12,29,35,36]. Sexual dimorphism can enhance pollination for both morphs, and generalist pollination strategies help maintain reproductive success in changing environments. The continuity and evolution of gynodioecious systems depend on the efficiency and availability of pollinators [37,38]. Therefore, it is critical to consider flower morphology, reproductive biology, and pollinator interactions together, especially in gynodioecious species with limited distribution, to understand the ecological resilience and evolutionary continuity of these reproductive systems.

The presence of both female and hermaphrodite flowers is common among members of the Caryophyllaceae family [13,39,40,41,42,43,44,45,46,47,48,49,50]. Some species are strictly gynodioic, while others are gynomonoic, which means they have individuals with both female and hermaphrodite flowers on the same plant [41,44,45]. Research on gynodioecy in the genus *Minuartia* is limited. In the *Minuartia rossii* complex (*M. austromontana*, *M. elegans*, and *M. rossii*), *M. austromontana* and *M. elegans* have been found to be both female and hermaphrodite and possess a complex reproductive strategy [51]. However, this complex was later removed from the genus *Minuartia* and placed under the genus *Sabulina* (Dillenberger and Kadereit, 2014) [52]. Currently, *Minuartia* is represented by 66 species, of which only *M. nifensis* is known to be gynodioic [53,54]. Of the 29 *Minuartia* species (38 taxa, including 24 endemic species, subspecies, and varieties) found in Turkey, *M. nifensis* is an endemic species with a very narrow distribution [55]. This combination of gynodioecy, taxonomic singularity within the genus, and restricted endemism makes *M. nifensis* a suitable model for studying the evolution, ecology, and function of gynodioecious reproductive systems.

This study aims to determine the distribution area and population size of *M. nifensis* and identify the effects of gynodioecy on this rare species. To accomplish this, we will evaluate key reproductive characteristics, such as the ratio of hermaphrodite to female morphs in the population, intermorphic flower morphology, pollen viability, stigma receptivity, self-incompatibility rates, seed-setting success, and pollinator diversity. Specifically, we test the following hypotheses: (H1) reproductive characteristics differ significantly between morphs and (H2) pollinator communities and visiting behaviors vary and affect seed-setting outcomes.

## 2. Results

### 2.1. Distribution Area, Number of Individuals and IUCN Category

**Figure 1:**
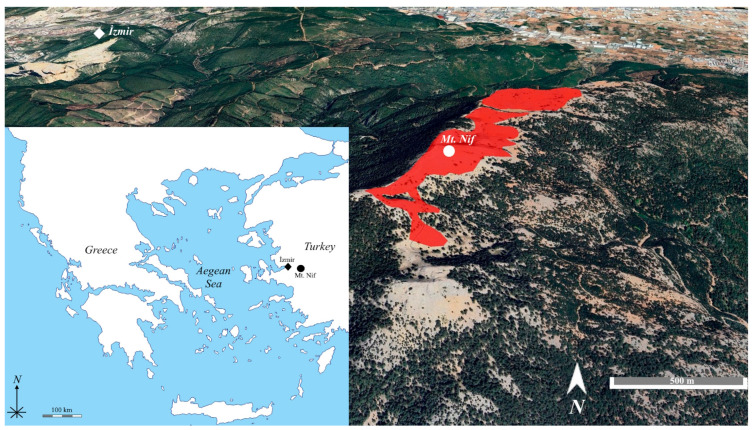
The distribution area of *M. nifensis*.

*Minuartia nifensis* is found in the subalpine region, where trees begin to become sparse. It grows in the cracks of limestone cliffs where the soil layer is almost absent, among gravel and amongst the surrounding flora. It has been observed that the seeds of this species hiding to the undersides of the leaves of other species, such as *Minuartia hirsuta* subsp. *asiatica*, *Thymus sipyleus*, *Paronychia argentea*, *Festuca pinifolia* and *Asperula daphneola*, in order to germinate while being protected from the effects of the wind. The species’ distribution area was found to be between 1375 and 1505 m, and when GPS records were used to plot its boundaries, the total area was found to be 0.52 km^2^ (Figure 1). Counts made by visiting every point in the total distribution area revealed that there were 4296 individuals of the species. Within the distribution area of *M. nifensis*, a total of 752 m^2^ under the authority of the Regional Directorate of Forestry includes a previously constructed fire observation tower, a radio transmission facility, and an access road serving these structures. Apart from these long-established infrastructure elements, no additional or ongoing anthropogenic pressures were recorded within the species’ range during the study period. However, the extremely restricted area of occupancy and the presence of infrastructure within the distribution area may represent potential future risks. Therefore, provided that further monitoring of the species is carried out, it may be considered for evaluation under the CR B2ab(iii) criterion of the IUCN red list categories.

### 2.2. Evaluating of Morph Ratios

**Figure 2:**
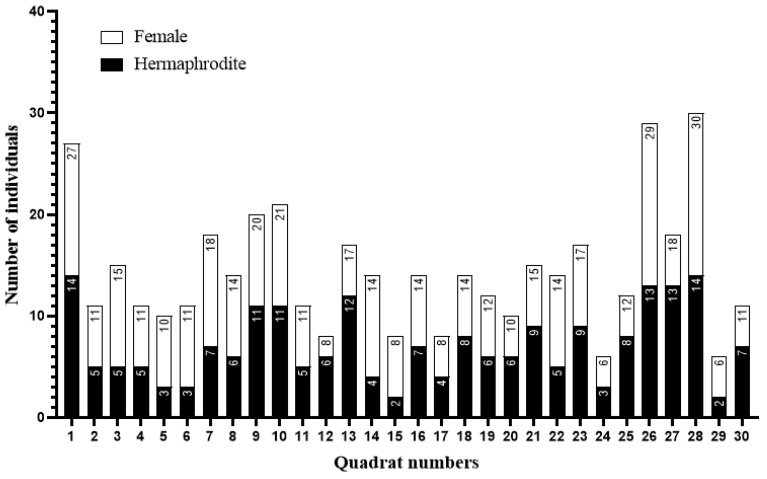
Sex ratio of *M. nifensis* in 30 quadrats. Differences between the numbers of hermaphrodite and female individuals recorded within the same quadrats were assessed using a Wilcoxon matched-pairs signed-rank test.

A total of 213 hermaphrodites and 219 females of *M. nifensis* were recorded in 30 quadrats. The hermaphrodite/female ratio in the population was approximately 0.97:1, and no significant imbalance was observed between the morphs. At the quadrat level, the median number of individuals was comparable between morphs (hermaphrodites: median = 7, range = 2–15; females: median = 7, range = 2–16). A Wilcoxon matched-pairs signed-rank test revealed no significant difference between the numbers of hermaphrodite and female individuals recorded within the same quadrats (*p* = 0.621). This balanced distribution was observed despite heterogeneity in the sampled microhabitats, suggesting that both sexual morphs exhibit similar tolerance to local environmental variation (Figure 2).

### 2.3. Comparison of Hermaphrodite and Female Flower Morphology

**Figure 3:**
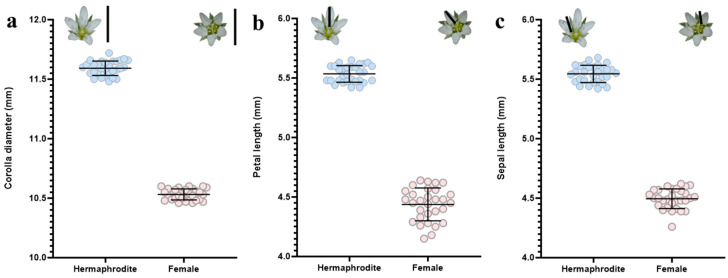
Morphometric measurements in hermaphrodite and female flowers. Observed data and models estimated means (±SD, in mm) for (**a**) corolla diameter, (**b**) petal length and (**c**) calyx length. Differences between morphs were analyzed using unpaired Student’s *t*-tests.

All measurements of floral size in hermaphrodite and female flowers (corolla diameter, petal length, calyx length) indicate that hermaphrodite flowers are significantly larger than female flowers (Figure 3a corolla diameter: t = 75.7, df = 58, *p* < 0.0001; Figure 3b petal length: t = 38.8, df = 58, *p* < 0.0001; Figure 3c sepal length: t = 52.2, df = 58, *p* < 0.0001). The exceptionally high t-values observed in these floral dimensions are attributable to the discrete, non-overlapping phenotypic separation between the two sexual morphs and the remarkably low intra-morph variation, confirming a clear sexual dimorphism in floral size.

### 2.4. Pollen Viability and Stigma Receptivity

**Figure 4:**
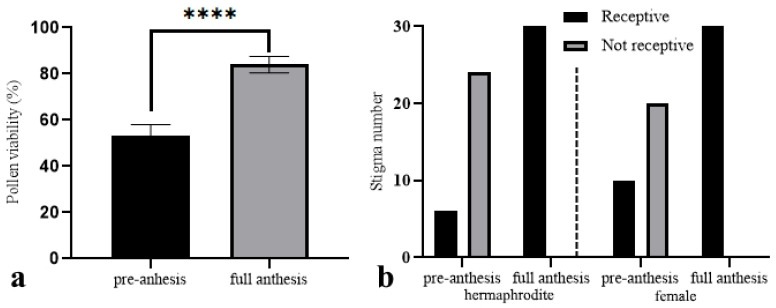
Pollen viability and stigma receptivity in *M. nifensis*. (**a**) Pollen viability in hermaphrodite flowers at pre-anthesis and full anthesis (unpaired *t*-test), **** indicates (*p* < 0.0001) statistical significance level. (**b**) Stigma receptivity in hermaphrodite and female flowers (χ^2^ test).

The mean pollen viability percentage recorded was 53 ± 5.05% in the pre-anthesis stage, and 84 ± 3.51% at full anthesis (Figure 4a). The variation between stages was found to be statistically significant (t = 27.61, df = 58, *p* = 0.0001; unpaired *t*-test). The mean difference in pollen viability was 31% with a 95% confidence interval ranging from 28.75% to 33.25%, indicating a strong phenological effect on pollen viability. In hermaphroditic flowers, the receptivity of the stigma increased from 20.0% at the pre-anthesis stage (six receptive out of 30) to 100% at full anthesis (30/30). In the case of female flowers, receptivity exhibited a marked increase from 33.3% at pre-anthesis (10/30) to 100% at full anthesis (30/30) (Figure 4b). The application of a contingency analysis revealed that these differences were statistically significant (χ^2^(3) = 70.62, *p* < 0.0001), indicating a strong effect of both phenological stage and sexual morph on stigma receptivity.

### 2.5. Temporal Details of Flower Anthesis

**Figure 5:**
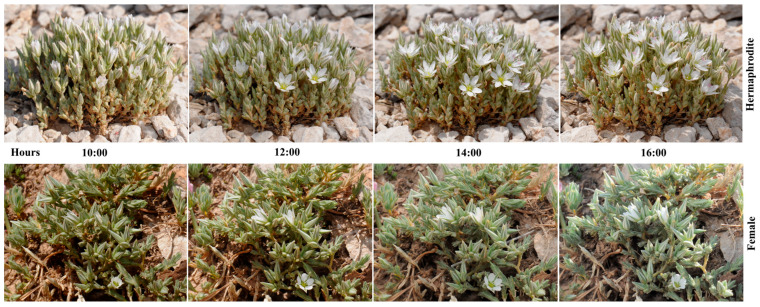
Anthesis period in hermaphrodite and female individuals of *M. nifensis*.

In both morphs, the onset of anthesis occurs at approximately 10:00. The flowers reach full anthesis between 12:00 and 14:00. Subsequent to 16:00, the flowers commence a process of closure once more, which is fully completed within a period of one and a half hours (Figure 5). It is evident from the observations that the optimal time for pollination during the day is the 6.5 h period between 10:30 and 17:00, i.e., between half an hour after the flower opens and half an hour before it closes. The lifespan of flowers is two days, after which the process of fruiting begins. On the first day, from the moment the flower begins to open, the five inner stamens sequentially release their pollen, and on the second day, the outer stamens also open sequentially and release their pollen. This strategy of releasing pollen from the anthers over two days, as instead of a single release, has been demonstrated to provide fresh pollen to pollinators during their visits throughout the flowering period, thus contributing to cross-pollination.

### 2.6. Pollen/Ovule Ratio and Index of Self-Incompatiblity in Hermaphrodites

The mean pollen count and mean ovule number in hermaphrodite individuals were determined to be 1380.5 ± 202.41 and 13.06 ± 2.75, respectively, resulting in a calculated P/O ratio of approximately 105.7. This value is consistent with mating systems in which selfing may occur but does not, by itself, indicate the predominance of autogamy.

The seed set in hermaphrodite individuals was determined to be 0.86 ± 1.5 after self-pollination and 5.34 ± 1.6 after cross-pollination. Consequently, the ISI is equivalent to 0.86/5.34, which is equivalent to 0.161. This low ISI value reflects substantially reduced reproductive success under self-pollination relative to cross-pollination.

### 2.7. Mating System

Significant differences in seed sets were found when the reproductive success of hermaphrodite and female morphs was evaluated (Table 1). The number of ovules in hermaphrodite flowers indicated that the plant could potentially produce 13.06 ± 2.75 seeds. When seed production under different pollination treatments was evaluated relative to ovule number, statistically significant differences were detected among open pollination, apomixis, autogamy, and allogamy experiments (*p* < 0.0001). In hermaphrodites, the potential seed production was found to be 16.69% in open pollination, 0% in apomixis, 6.58% in autogamy and 40.88% in allogamy. The difference in seed set between open pollination and apomixis/allogamy was again quite high (*p* < 0.0001), the difference between open pollination and autogamy was statistically significant (*p* = 0.0013), although the magnitude of the difference was smaller than that observed for other comparisons (*p* = 0.0013). The lack of any seed set in the apomixis detection experiment indicated that hermaphrodite individuals had only sexual reproduction. As the seed set resulting from autogamy was low, the results were similar to those of the apomixis experiment and no significant difference was observed between them (*p* = 0.092). The difference in seed set between autogamy and allogamy was found to be significant (*p* < 0.0001). In conclusion, the experiments showed that hermaphrodite individuals do not use apomixis for reproduction. However, they can reproduce by autogamy at a low rate and depend on the allogamous reproductive strategy, which requires the presence of a pollinator. Since pollen was not produced in the female individuals’ anthers, all experiments except autogamy were performed. The potential seed-producing capacity of the female individuals was determined to be 13.58 ± 2.7 ovules. According to the potential for seed production in females, the success rates were found to be 15.32% for open pollination, 0% for apomixis, and 45.95% for allogamy. When the results of all the trials involving females were compared, it was found that there was a significant difference between them (*p* < 0.0001). All trials in females depended on allogamy only in the presence of a pollinator.

**Figure 6:**
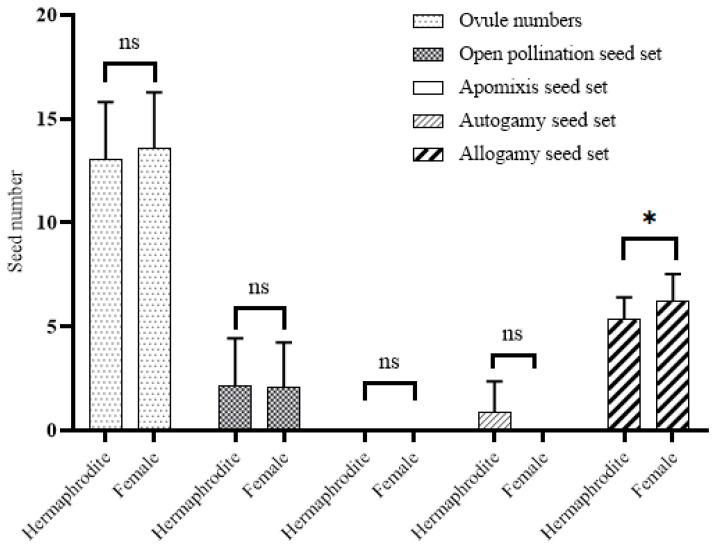
Comparison of reproductive system success among morphs [n = 50 hermaphrodite and female]. Data were analyzed using two-way ANOVA followed by Sidak’s multiple comparisons test. Asterisks indicate levels of statistical significance [ns (not significant), * (*p* < 0.05)].

Similar levels of reproductive capacity were generally observed between individual morphs when ovule production was compared, while seed yield differed depending on pollination route (Figure 6). There was no significant difference in ovule number between the two sex morphs, indicating that potential reproductive capacity, as measured by ovule production, is independent of sex. Similar results were obtained for seed numbers under open pollination, with no significant difference found between the two morphs. In apomixis experiments, no seed formation was observed in either morph, indicating that apomictic reproduction does not occur in this species. In autogamy experiments, seed formation was only observed in hermaphrodite individuals, as expected; this route was not observed in female individuals. Seed formation due to autogamy was observed only in hermaphrodite individuals; however, this difference was not statistically significant (*p* = 0.0619). This suggests that autonomous self-pollination is possible, albeit to a limited extent. In cross-pollination (allogamy) experiments, a statistically significant difference was found between the two morphs. Female individuals produced more seeds than hermaphrodites, and this difference was statistically significant (*p* = 0.0448). This finding suggests that female individuals may have higher seed yields through cross-pollination.

Overall, both individual morphs exhibited the highest seed success under external pollination (allogamy). Seed numbers obtained through other means, particularly with alternative strategies such as apomixis and autogamy, were quite limited. These data suggest that the species is highly dependent on external pollination for effective seed production.

### 2.8. Floral Visitors and Their Behaviors

**Figure 7:**
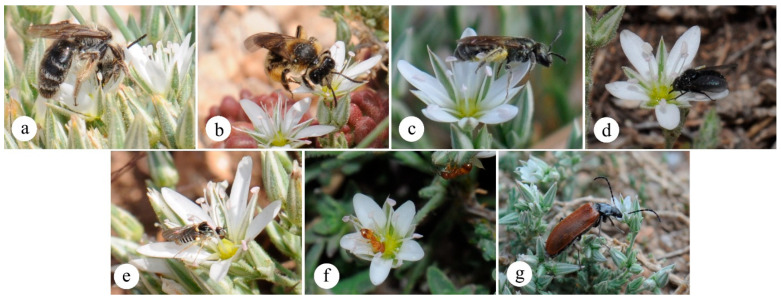
Flower visitors of *M. nifensis* (**a**) *Andrena alutacea* (**b**) *Andrena flavipes* (**c**) *Lasioglossum* sp. (**d**) *Usia* sp. (**e**) *Cephalodromia* sp. (**f**) *Themnothorax* sp. (**g**) *Omophlus lepturoides*.

**Figure 8:**
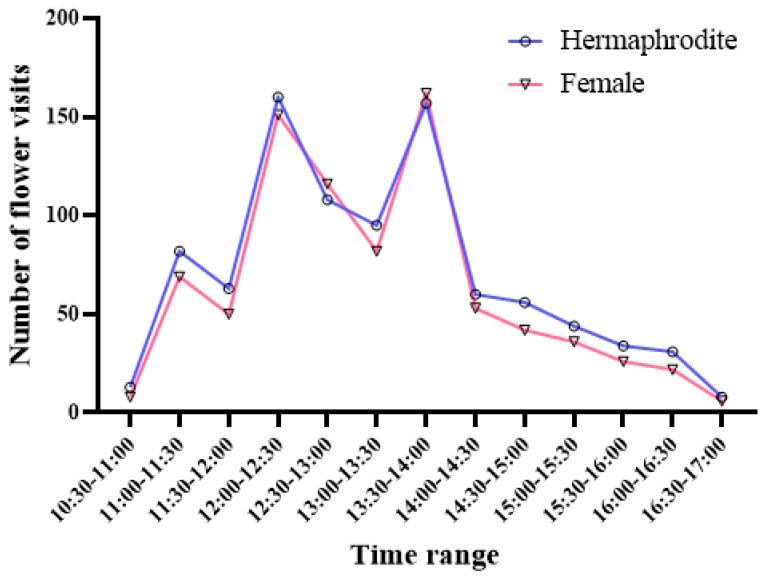
Time-dependent flower visitor numbers of hermaphrodite and female flowers of *M. nifensis*. Values represent descriptive visitation frequencies.

The observations of *M. nifensis* flower visitors revealed that the flowers were visited a total of 1734 times by 7 visitors belonging to 6 different families (Figure 7). Of the visitor species, three collected pollen and nectar, three collected only nectar, and one was a predator species that arrived to consume the flower. With regard to inter-individual pollen transfer, only *Andrena alutacea*, *A. flavipes*, and *Lasioglossum* sp. were observed to be effective, particularly with pollen carried in the scopa and ventral regions, accounting for 95% of the total visits. The species *Usia* sp. and *Cephalodromia* sp. were observed to collect nectar using their stylets without making contact with the anthers. In contrast, *Themnothorax* sp. exhibited an absence of pollen attachment to its body even during movement across the flower, and this species was solely engaged in nectar collection. Conversely, *Omophlus lepturoides* has been observed to consume all components of the flower except the sepals. In *M. nifensis*, flowers receive the greatest number of visits (58% of total visits) between 12:00 and 14:00 h, during periods of wind speeds below 1 m/s. The simple linear regression analysis revealed a significant negative relationship between wind speed and total pollinator visits (*R*^2^ = 0.66, *F*(1.11) = 20.98, *p*< 0.001). The model indicates that for every 1 m/s increase in wind speed, the number of visits decreased by an average of 74.19 (*β* = −74.19, 95% Cl: −109.9 to −38.53). Of the total visits, 52.54% (911 visits) were to hermaphrodite flowers and 47.46% (823 visits) were to female flowers. With the exception of the peak visitation period between 12:00 and 14:00, visits to female flowers were observed to be comparatively lower (Figure 8).

## 3. Discussion

Gynodioecious plants are defined as those in which the relative ratio of female to hermaphrodite individuals within a population is considered a key parameter reflecting the evolutionary balance between reproductive morphs and the long-term stability of the system. Research has indicated that the frequency of females is typically low, exhibiting significant variation among populations within specific species. In some cases, ratios have been observed to approach 1:1, suggesting a potential shift towards dioecy [28,56]. The approximately equal hermaphrodite–female ratio may indicate a relatively balanced reproductive structure within the studied population. However, evaluating the long-term stability of this system would require data from multiple populations and multiple years. Theoretical and experimental studies have demonstrated that for females to maintain their presence in the population, it is imperative that they attain a substantial advantage in seed fitness when compared to hermaphrodites. In the absence of such a significant advantage, the loss of male function will be eradicated through selection processes [56,57]. In this study, the elevated seed production exhibited by female individuals under the context of allogamy serves as the underlying mechanism that elucidates the balanced sex ratio observed in *M. nifensis*. This finding underscores the notion that the female advantage is sufficiently robust to perpetuate polymorphism. Furthermore, the ratio close to 1:1 indicates an equilibrium state where female frequency does not push pollen limitation to a critical threshold, while hermaphrodites maintain high male fitness through pollen export. This pattern is compatible with theoretical expectations of cytoplasmic male sterility–nuclear restorer systems [58]; however, no genetic data are currently available to test this mechanism in *M. nifensis*. These interpretations should therefore be regarded as hypotheses rather than demonstrated mechanisms. Robust evaluation of evolutionary stability in *M. nifensis* would require population-genetic analyses, identification of CMS cytotypes and nuclear restorer loci, and comparative data across multiple populations and years.

The statistically significant differences observed in corolla diameter, petal length, and sepal length between hermaphrodite and female individuals in *M. nifensis* strongly correlate with sex-specific flower morphology patterns commonly reported in gynodioecious plants. All three traits were larger in hermaphrodite individuals. This pattern may indicate that the evolution of floral structures was primarily driven to support male function, particularly pollen export and pollinator attraction. Research on this topic emphasizes that larger corolla diameters and longer petals enhance visual signals, increasing the frequency and effectiveness of pollinator visits. Conversely, the reduction in these costly structures in non-pollen-producing female flowers allows resources to be directed towards ovule, fruit, and seed development [24,59,60]. The reduction in corolla size and petal length in female flowers indicates reduced investment in pollinator attraction. In the absence of male function, female fitness is primarily optimized through enhanced seed production efficiency. Furthermore, the observation of elongated sepals in hermaphrodites suggests the presence of protective and structural requirements associated with larger stamens and higher pollen loads. Conversely, the shorter sepals in females appear to reflect a more compact gynoecium architecture, which may be compatible with limited resources and efficient in terms of resource allocation. These results suggest that perianth dimensions reflect the functional demands of the reproductive organs. They also indicate that sex-specific selection pressures may influence the evolution of floral morphology [61]. Therefore, the morphological dimorphism identified in *M. nifensis* clearly reveals the evolutionary equilibrium in gynodioic systems. In such systems, hermaphrodites gravitate towards a more showy flower morphology that supports male function, while females gravitate towards a simpler and more cost-effective flower morphology that enhances female fitness.

In numerous plant species, the onset of flowering signifies the maximum extent of pollen viability and the commencement of stigma receptivity, thereby facilitating pollination. Concurrently, flower closure typically transpires subsequent to pollination or as pollen viability and stigma receptivity undergo a decline. Consequently, reproductive success is optimized, while energy expenditure is minimized. For instance, within the Gesneriaceae family, pollen germination rates attain a maximum of 1 to 9 days following flowering, contingent on the specific species, with the most substantial pollen tube growth occurring within the initial 1–3 days [62]. In a similar manner, in *Adenium obesum*, pollen viability has been shown to exceed 69% at temperatures ranging from 25 to 30 °C from the onset of flowering onwards, thereby enabling effective in vitro germination of up to 39.81% under optimal conditions [63]. The temporal coincidence of these events is not coincidental; flowering is driven by cell expansion, which is fueled by osmotic changes such as the conversion of starch or fructose into monosaccharides. These changes, in turn, promote pollen maturation and release [64,65]. The period of receptivity to stigma typically commences immediately after or shortly after flowering, thereby establishing a temporal window conducive to successful fertilization. In *Moringa oleifera*, the opening of flowers occurs during the early morning hours (6:00–12:00), followed by the release of pollen (7:00–13:00). This temporal pattern of pollination is hypothesized to be a mechanism that delays stigma receptivity, thereby promoting cross-pollination [66]. The presence of biochemical markers, including esterases and additional proteins, has been observed to increase stigma receptivity at this stage, as evidenced by histochemical tests [66]. In the genus *Wittmackia*, both pollen viability and stigma enzymatic activity reach their peak during the flowering period [67]. In *M. nifensis*, stigma receptivity gradually increases during flower development and reaches its highest levels during the full anthesis. Similarly, pollen viability in hermaphrodite flowers increases from relatively low levels during pre-anthesis (53%) to high levels during full anthesis (84%). With respect to the temporal characteristics of flowering, it was ascertained that the period of full anthesis occurs during a 2 h interval (specifically between 12:00 and 14:00) of the 6.5 h cycle encompassing the opening and closing of the flowers. This specific time frame coincides with the period of peak insect activity, accounting for 58% of the total visits. This finding indicates a close temporal correspondence between the opening of flowers, reproductive traits, and pollinator activity in *M. nifensis*, thus demonstrating that the 2 h full anthesis period during the day is a critical period for the reproduction of *M. nifensis*.

In gynodioic systems, the fundamental mechanisms ensuring the sustainability of female individuals in the population are the compensation for the genetic disadvantage caused by male sterility. Meta-analysis studies have indicated that the superiority of female morphs over hermaphrodites in terms of seed number contributes to the conservation of female morphs [68]. The absence of a statistically significant difference in ovule numbers between female and hermaphrodite individuals in *M. nifensis* indicates that male sterility in the species does not directly impact ovule production. This finding aligns with the observations made in studies of *Geranium sylvaticum*, a gynodioic species in which ovule production does not exhibit a direct correlation with sex [56,69]. However, the markedly lower seed set observed under autogamy compared to cross-pollination indicates that self-fertilization is limited in *M. nifensis*. Experimental pollination results, quantified using the index of self-incompatibility (ISI), indicate that seed set following self-pollination is markedly lower than that obtained through cross-pollination, suggesting a strong reliance on outcrossing in hermaphrodite individuals. It has been observed that the seeds produced through autogamy in angiosperms exhibit a lower seed yield compared to those produced through allogamy [70]. However, the limited seed production observed under autogamy in some hermaphrodite individuals may contribute marginally to reproductive assurance under conditions of reduced pollinator availability, although this pathway appears to play a secondary role compared to cross-pollination. Apomixis trials indicate the absence of apomictic reproduction in *M. nifenis*, a finding consistent with the observation that apomixis is subject to stringent regulation by specific genetic and developmental mechanisms in numerous plant species [71]. The findings suggest that successful seed production in *M. nifensis* is largely associated with pollinator-mediated cross-pollination, as reflected by the significantly higher seed formation rates observed both sex morphs in allogamy (cross-pollination) trials. A similar phenomenon has been observed in *Dianthus sylvestris*, which exhibits both gynodioecious and gynomonoecious characteristics. Studies have demonstrated that female individuals achieve higher seeding rates from cross-pollination, thereby contributing to a female advantage and the maintenance of sex balance in the population [41]. Consequently, the observation that cross-pollination enhances the seed set in both female and hermaphrodite individuals in *M. nifensis* indicates that genetic sustainability might be elevated in natural populations of the species in the absence of pollinator limitations. In flowering plants, approximately 20% of hermaphrodite species are fully self-fertilizing, while approximately 33% utilize mixed mating systems [72,73]. Taken together, these findings indicate that *M. nifensis* exhibits a mixed mating system, in which cross-pollination constitutes the primary and most successful reproductive mode, while limited self-fertilization in hermaphrodite individuals may function as a secondary mechanism providing reproductive assurance. This system provides female individuals with a higher reproductive advantage compared to hermaphrodites in the presence of a pollinator. Conversely, limited self-fertilization in hermaphrodite individuals may provide reproductive assurance under conditions of reduced pollinator availability, although cross-pollination clearly represents the primary and more successful reproductive pathway.

In gynodioecious plant species, pollinator visitation frequently differs between hermaphrodite and female flowers due to sex-specific variation in floral display and reward structures, often resulting in reduced visitation to female flowers [74,75]. In *M. nifensis*, the overall visitation rates exhibited relative balance between floral morphs, with 52.54% of visits directed to hermaphrodite flowers and 47.46% to female flowers. This pattern stands in contrast to the strong pollinator discrimination against females reported for *Geranium richardsonii* and *G. maculatum*, where female flowers typically receive approximately half the visitation rate of hermaphrodites [74,75]. It is noteworthy that in *M. nifensis*, this near-parity in visitation was primarily observed during the peak activity window, which occurred between 12:00 and 14:00, when wind speed was low (<1 m s^−1^). However, outside this period, female flowers experienced comparatively reduced visitation. This temporal pattern suggests that pollinator discrimination in *M. nifensis* is context dependent rather than constant, aligning with frequency- and environment-dependent pollinator behavior described by Van Etten and Chang [75]. Furthermore, although a diverse assemblage of visitors was recorded, the majority of floral visits were performed by three bee taxa (*Andrena alutacea*, *A. flavipes*, and *Lasioglossum* sp.), accounting for 95% of total visits. Based on their frequent contact with reproductive organs during foraging, these taxa are considered potentially effective pollinators. This finding indicates that most pollinator activity is concentrated in a narrow subset of visitors. In accordance with observations made in the context of *Geranium* species, non-pollinating or weakly pollinating visitors (e.g., nectar robbers or predators) are likely to contribute marginally to female reproductive success despite their frequent visits [74]. The relatively high visitation to female flowers during optimal conditions in *M. nifensis* may therefore mitigate the fitness disadvantages typically associated with reduced floral attractiveness in females. This results suggest that gynodioecy in this species is not maintained by strong pollinator bias toward hermaphrodites. Instead, it is depend on temporally structured pollinator activity and frequent visitation by a limited group of likely pollinators that consistently contact reproductive organs. This finding underscores the notion that pollinator discrimination against female flowers, while prevalent in gynodioecious systems, is not an invariable phenomenon. It is plausible that this discrimination may be mitigated in systems where the interplay of floral morphology, visitor behavior, and environmental conditions collectively enable adequate pollen delivery to female morphs.

Although this study focused on empirically testable differences in reproductive traits and pollination dynamics, the observed variation in self-compatibility between morphs also has potential implications for population persistence under fluctuating pollination environments. Hermaphrodite individuals provide reproductive assurance through self-compatibility, whereas female individuals depend entirely on effective cross-pollination. While these contrasting strategies may influence long-term population dynamics, such effects were not directly evaluated here and would require explicit demographic modeling and temporal data to be tested. Similarly, the consistent morphological, physiological, and functional differentiation observed between hermaphrodite and female individuals is unlikely to reflect random variation or incomplete sexual differentiation alone. Instead, these patterns are consistent with selective pressures commonly associated with dioecy-related reproductive strategies in gynodioecious systems. However, direct evidence for such selection was not assessed in this study. Future research integrating population-level comparisons across sites and genetic approaches, including tests for cytoplasmic male sterility (CMS) and nuclear restorer dynamics, would be necessary to evaluate the evolutionary mechanisms underlying the maintenance of gynodioecy in *M. nifensis*.

## 4. Materials and Methods

### 4.1. Plant Species and Study Area

*Minuartia nifensis* is a perennial plant that distributed on open rocky and gravelly places in the alpine regions at approximately 1250 m altitudes of the Mount Nif, South of the İzmir city center, and is a local endemic species with a single population [76]. The study was carried out between June 2013 and July 2025 in Mt. Nif, the only known locality in the Aegean Region of Turkey (38°23′16.85″ N, 27°21′23.45″ E). The mean annual temperature in this region is 22.5 °C (min: 11 °C, max: 35 °C), and the annual mean rainfall is around 1050 mm [77].

### 4.2. Distribution Area and Number of Individuals

The lower and upper limits of the distribution of *M. nifensis* were determined using ‘Magellan Explorist 500’ GPS (Magellan Navigation, Inc., San Dimas, CA, USA) records. These records were processed in Google Earth Pro version 7.3.6 to create a distribution map and define the species’ distribution areas. Individuals were counted through direct observation throughout the determined distribution area. Based on the data obtained, the species was evaluated according to the “IUCN Red List categories” and version 3.1 criteria.

### 4.3. Determination of Morph Ratios

**Figure 9:**
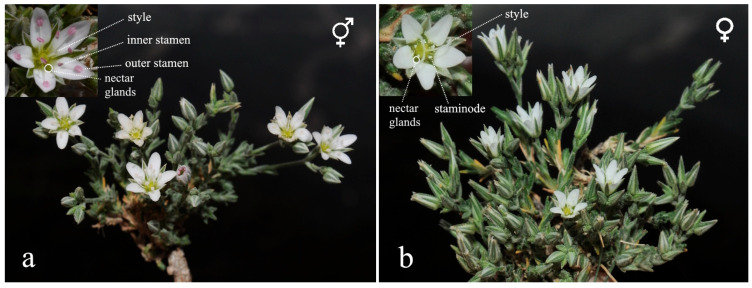
Flower structures of *M. nifensis* (**a**) hermaphrodite; (**b**) female.

To determine individual morph ratio, thirty 5 m × 5 m sampling quadrats were established within the species’ known distribution area. Quadrat locations were selected heterogeneously to reflect the topographic and microhabitat diversity of the study area and were randomly distributed throughout the area. Only flowering and reproductively mature (adult) individuals were considered within each quadrat. Individuals were counted and classified as hermaphroditic or female based on the morphological characteristics of their floral structures (Figure 9).

### 4.4. Quantitative and Functional Analysis of Floral and Reproductive Traits

In order to examine the morphological and physiological characteristics of the reproductive system, detailed measurements were carried out on one hermaphrodite and one female individual randomly selected from each of the 30 individual 5 × 5 m quadrats used in the study [n = 30 hermaphrodite individuals, n = 30 female individuals]. Collected flowers preserved in formalin Acetic Alcohol (FAA: acetic acid 5%, formaldehyde 5%, and ethanol 90%) solution and transported to the laboratory. Morphological measurements of corolla diameter, petal length, sepal length in one fully developed flower were performed on each individual using a Dino-Lite AM7025X camera (Dino-Lite Europe/IDCP B.V., Almere, The Netherland). One sample of mature capsules was taken from different flowers of the same individuals and the number of ovules per capsule was counted using an Olympus SZ61 stereo microscope (Olympus Corporation, Tokyo, Japan).

To determine pollen production in one anther per flower in hermaphrodite individuals, unopened anther was transferred to Eppendorf, 0.9 mL of ethanol, 4 drops of 1% methylene blue solution and 4 drops of Tween 20 were added and crushed. The solution was centrifuged for 60 s at 2800 rpm in the Biosan FV-2400 Micro-spin (SIA Biosan, Riga, Latvia) and the supernatant was transferred to the Thoma cell counting chamber (Isolab, İstanbul, Türkiye) and the pollen grains were counted. Since hermaphrodite individuals have 10 stamens, the number of pollen produced per flower was calculated by multiplying the number of pollen in an anther by ten.

The viability of pollen and the receptivity of stigma were assessed using samples taken from pre-anthesis [n = 30 flowers] (just before anthesis, flowers that have not yet opened) and post-anthesis [n = 30 flowers] (fully opened flowers) stages. Pollen viability was determined by dehydrogenase activity test, and stigma receptivity was determined by peroxidase activity test. Tests were carried out at 30 °C. For the detection of dehydrogenase presence, MTT (3-(4,5-Dimethyl-2-thiazolyl)-2,5-diphenyl-2H-tetrazolium bromide, Sigma M-2128, 10 mg) was dissolved in a 5% sucrose solution and stored as stock solution at 2–8 °C in the dark. pollen samples were placed on a slide with 5–10 μL of the reactive droplet; the drying of the reactive was awaited, and the process was repeated three times. After 10 min, pollen grains that stained dark purple were considered to have dehydrogenase activity [78]. For the detection of peroxidase activity, 3,3′-Diaminobenzidine tablets (Sigma, D4293) were dissolved in 1 mL of distilled water in an Eppendorf tube to prepare the reactive solution. Stigma samples were immersed in 1 mL of the reactive solution. After 5 min, stigmas and pollen grains that stained brown were considered to have peroxidase activity [79].

### 4.5. Experimental Investigation of the Mating System

To measure natural levels of seed production in both morphs as part of the reproductive system assessments, a total of 200 hermaphrodites and 150 female individuals, at least 1 individual from each of 30 quadrats under controlled conditions, were randomly selected, labeled, and bagged with waxed white paper. Bagging and pollen supplementation experiments were conducted to examine the possibility of apomixis, autogamy and allogamy. The following treatments were applied to the flowers of hermaphrodite individuals; (i) for open pollination, flowers were left open to visit by pollinators [n = 50 flowers], (ii) for apomixis experiment, stamens were emasculated and flowers were bagged [n = 50 flowers], (iii) for the autogamy experiment, flowers pollinated with the flower’s own pollen were bagged [n = 50 flowers], (iv) for the allogamy experiment, the male organs of the flower used as the mother were emasculated and pollinated with pollen from different hermaphrodite flowers and bagged [n = 50 flowers]. The following treatments were applied to the flowers of female individuals; (i) for open pollination, flowers were left open to visit by pollinators [n = 50 flowers], (ii) for the apomixis experiment, flowers were directly bagged [n = 50 flowers], (iii) for the allogamy experiment, pollinated with pollen from hermaphrodite flowers and bagged [n = 50 flowers]. Two weeks after the experimental set was established, the capsules were collected and the seeds in each capsule were counted.

### 4.6. Determination of Pollen:Ovule Ratio and Self-Incompatibility in Hermaphrodites

In order to gain further insights into the breeding system of the hermaphroditic flowers, the pollen/ovule (P/O) ratio was calculated by dividing the total number of pollen grains (counted from all anthers of a flower) by the number of ovules in the same flower. The resulting ratio was interpreted according to the classification system developed by Cruden [80], in which P/O values of 2.7–5.4 are indicative of cleistogamy, 8.1–39 of obligate autogamy, 31.9–396 of facultative autogamy, 244.7–2588 of facultative xenogamy, and 2108–195,525 of obligate xenogamy. As female individuals do not produce pollen, the P/O ratio was not calculated for them and was applied only to hermaphroditic individuals.

In order to evaluate the breeding system of the species, the self-incompatibility index (ISI) was calculated for hermaphroditic individuals using the equation proposed by Zapata and Arroyo [81]. The index of self-incompatibility (ISI) was calculated as the ratio of seed set obtained from self-pollination to that obtained from cross-pollination (ISI = selfed seed set/cross-pollinated seed set). According to this classification, an ISI value of 1 indicates complete self-incompatibility, values between >0.2 and <1 indicate partial self-incompatibility, and values <0.2 indicate mostly self-incompatibility. As female individuals are characterized by their functional unisexuality and absence of stamens, they are incapable of producing pollen and consequently cannot undergo self-pollination. Consequently, ISI values were exclusively calculated for hermaphroditic individuals.

### 4.7. Floral Visitors and Their Behaviors

Data on the floral visitors of *M. nifensis* were collected through direct observations conducted in June, during the peak flowering period, on sunny days with low to moderate wind conditions. Observations were carried out in three 25 m^2^ quadrats (5 m × 5 m) distributed at random within distribution areas. Each quadrat contained ten mature individuals. Based on preliminary observations conducted on three different days between 06:00 and 20:00, the effective pollination hours, which are the start and end times of pollinator visits, were determined to be 10:30–17:00. Observers remained 1 m away from the quadrats to avoid disturbing pollinator activity. Each quadrat was observed for six hours per day over four consecutive days, with observations recorded at half hour intervals between 10:30 and 17:00. Wind speed and temperature were also recorded during each half hour observation. The hourly mean values of wind speed and temperature were then calculated for each based on data from the three quadrats (each repeated twice) for the preparation of tables (Supplementary Data Table). In total, 72 h of observations were performed. The following data were collected within each quadrat: the identity of floral visitors observed on per flower; the number of per flower visited by each visitor; and whether each visitor exhibited behavior related to nectar foraging, pollen collection, or floral/bud predation.

### 4.8. Statistical Analysis

Differences in the number of hermaphrodite and female individuals recorded within the same quadrats were evaluated using a Wilcoxon matched-pairs signed-rank test, with quadrats treated as paired sampling units. The viability of pollen was determined exclusively in hermaphroditic flowers, with the proportion of viable pollen grains per flower being calculated at the pre-anthesis and full anthesis stages. The mean viability values between stages were then compared using an unpaired Student’s *t*-test. The study evaluated stigma receptivity in both hermaphroditic and female flowers. This was achieved by recording the number of receptive and non-receptive stigmas at the pre-anthesis and full anthesis stages. The analysis of these binary categorical data was conducted using the Chi-square test (χ^2^) to assess the existence of any disparities in receptivity between floral stages and sexual morphs. Fisher’s exact test was applied in instances where expected frequencies were less than five. Ovule number was analyzed separately as a pre-fertilization reproductive trait and was not included within the pollination treatment factor. Differences in ovule number between sexual morphs were analyzed independently as a structural reproductive trait using an unpaired Student’s *t*-test. To assess reproductive success, seed production under different pollination treatments (open pollination, apomixis, autogamy, and allogamy) was analyzed using a two-way ANOVA. In this model, sexual morph and pollination treatment were defined as fixed factors, while the number of seeds per capsule was used as the primary response variable. Prior to analysis, normality and homogeneity of variance assumptions were assessed using the Shapiro–Wilk and Levene’s tests, respectively. When the ANOVA revealed significant differences among reproductive models (*p* < 0.05) or significant interactions, Tukey’s Hones Significant Difference (HSD) or Sidak’s multiple comparisons test were used post hoc to determine pairwise differences between and within groups. Floral morphometric comparisons were based on measurements from 30 hermaphrodite and 30 female individuals (one flower per individual) using unpaired Student’s *t*-tests, were each floral trait served as a separate response variable. Mean differences, 95% confidence intervals (95% CI), and adjusted *p*-values were reported. A significance level of α = 0.05 was used in all tests. Statistical significance levels are shown in the graphs with the symbols * (*p* < 0.05), ** (*p* < 0.01), *** (*p* < 0.001) and **** (*p* < 0.0001). To evaluate the relationship between wind speed (m/s) and the total number of flower visits, a simple linear regression analysis was performed. In this model, wind speed was defined as the independent variable, while the total number of visits served as the dependent variable. The slope of the regression line and the coefficient of determination (R^2^) were used to assess the strength and direction of the relationship, with statistical significance determined at the 95% confidence level.

All statistical analyses were conducted using GraphPad Prism version 8.0 (GraphPad Software Inc., San Diego, CA, USA).

## 5. Conclusions

Our results demonstrate that gynodioecy in the narrowly endemic *M. nifensis* is maintained through pronounced and non-random differentiation between hermaphrodite and female individuals in morphological, physiological, and functional reproductive traits. Hermaphrodite individuals exhibit larger floral dimensions, functional pollen production with high viability, and reproductive flexibility through self-compatibility and multiple pollination pathways. In contrast, female individuals are fully specialized toward female function, lacking functional pollen and relying exclusively on cross-pollination for successful seed production, despite exhibiting stigma receptivity comparable to that of hermaphrodites. This clear division of reproductive roles indicates functional specialization rather than incomplete sexual differentiation.

Pollinator observations revealed a diverse assemblage of floral visitors and temporal variation in visitation activity, with no strong evidence of pollinator exclusion between morphs. Although hermaphrodite flowers received slightly higher visitation frequencies, seed production was consistently highest under open pollination, suggesting that pollinator-mediated pollen transfer enhances fertilization success under natural conditions. However, variation in visitation frequency between morphs did not translate into significant differences in seed set, indicating that the effects of pollinators on reproductive output can only be inferred indirectly.

Differences in self-compatibility between morphs further highlight contrasting mating system strategies, with hermaphrodites providing reproductive assurance under variable pollination environments and females depending entirely on outcrossing. While these patterns are consistent with selective pressures associated with dioecy-related reproductive strategies, the long-term evolutionary orientation of the population was not directly assessed and requires explicit demographic and evolutionary analyses.

Overall, the integration of floral trait differentiation, mating system variation, and pollinator-mediated pollen transfer underscores the functional stability of gynodioecy in *M. nifensis*. By identifying the distinct reproductive roles of hermaphrodite and female individuals, as well as their contrasting dependence on pollination pathways, this study provides a mechanistic framework for understanding how reproductive balance is maintained in small and spatially restricted populations.

From a conservation perspective, our findings contribute by highlighting key biological processes that are likely to influence population persistence in *M. nifensis*. The demonstrated reliance of female individuals on effective cross-pollination and the limited pollinator assemblage involved in pollen transfer indicate that disruptions to pollinator communities or flowering synchrony may disproportionately affect reproductive success and sex ratio stability. By clarifying the reproductive constraints and vulnerabilities inherent to this gynodioecious system, our results provide a biological basis for conservation assessments and emphasize the importance of maintaining pollinator availability and habitat conditions that support successful pollination in endemic plant populations.

## Figures and Tables

**Table 1:** A comparison of the success rates of reproductive systems in hermaphrodite and female individuals. Statistical significance levels showed as [ns (not significant), ** (*p* < 0.01) and **** (*p* < 0.0001)].

Hermaphrodite	Mean Difference	95.00% CI of Difference	Significant	Summary	Adjusted *p* Value
O vs. OP	10.88	9.939 to 11.82	Yes	****	<0.0001
O vs. ApS	13.06	12.12 to 14.00	Yes	****	<0.0001
O vs. AuS	12.2	11.26 to 13.14	Yes	****	<0.0001
O vs. AlS	7.72	6.779 to 8.661	Yes	****	<0.0001
OPS vs. ApS	2.18	1.239 to 3.121	Yes	****	<0.0001
OPS vs. AuS	1.32	0.3785 to 2.261	Yes	**	0.0013
OPS vs. AlS	−3.16	−4.101 to −2.219	Yes	****	<0.0001
ApS vs. AuS	−0.86	−1.801 to 0.08147	No	ns	0.092
ApS vs. AlS	−5.34	−6.281 to −4.399	Yes	****	<0.0001
ApS vs. AlS	−4.48	−5.421 to −3.539	Yes	****	<0.0001
**Female**	**Mean Difference**	**95.00% CI of difference**	**Significant**	**Summary**	**Adjusted** ***p*** **Value**
O vs. OPS	11.5	10.56 to 12.44	Yes	****	<0.0001
O vs. ApS	13.58	12.64 to 14.52	Yes	****	<0.0001
O vs. AlS	7.34	6.399 to 8.281	Yes	****	<0.0001
OPS vs. ApS	2.08	1.139 to 3.021	Yes	****	<0.0001
OPS vs. AlS	−4.16	−5.101 to −3.219	Yes	****	<0.0001
ApS vs. AlS	−6.24	−7.181 to −5.299	Yes	****	<0.0001

Ovule numbers: **O**, Open pollination seed set: **OPS**, Autogamy seed set: **AuS**, Allogamy seed set: **AlS**, Apomixis seed set: **ApS**.

## Data Availability

The data is archived at the Ege University Botanical Garden and Herbarium Research and Application Center and is available upon request.

## Supplementary Materials

The following supporting information can be downloaded at: https://www.mdpi.com/article/10.3390/plants15060913/s1.

## Abbreviations

The following abbreviations are used in this manuscript:

IUCN	International Union of Conservation Nature
ISI	Index of Self-Incompatibility
P/O	Pollen/Ovule ratio
